# Systematic implementation of cardiopulmonary ultrasound imaging to optimize management of acute decompensated heart failure

**DOI:** 10.1186/s43044-024-00529-8

**Published:** 2024-08-06

**Authors:** Ahmad Samir, Doaa Yosry, Ahmed Talaat Elgengehe, Kareem Said

**Affiliations:** Faculty of Medicine, Cairo University, Cairo, Egypt

**Keywords:** Lung ultrasound (LUS), Cardiopulmonary ultrasound imaging (CPUSI), Heart failure (HF), Filling pressures, 8-Zone, Pulmonary congestion

## Abstract

**Background:**

Heart failure (HF) poses a major health problem, where frequent HF rehospitalizations (HFH) heavily burden national health systems. HFH are predominantly linked to inadequate decongestion before discharge. It is uncertain if systematic implementation of cardio-pulmonary ultra-sound imaging (CPUSI) to standard HF management can improve outcomes and reduce HFH.

**Results:**

This study recruited 50 patients admitted with acute decompensated heart failure (ADHF). Besides the conventional daily assessment, CPUSI was systematically performed to guide treatment decisions, focusing on ventricular filling pressure and 8-zone lung ultrasound (LUS) score. On-admission and predischarge LUS scores were correlated to clinical outcomes. The mean age of the study group was 55.7 ± 10.59 years, with predominance of male gender. Supplementing clinical judgment, CPUSI modified therapeutic strategy in 57 out of 241 assessments (24%), improving patients’ care. Besides its value in guiding therapeutic decisions, the LUS score on admission had a significant positive correlation to the length of ICU stay and the total hospitalization length. Also, LUS score > 12 at discharge predicted 90-day HFH with sensitivity and specificity of 100% and 98%, respectively.

**Conclusions:**

Systematic CPUSI can improve HF management by complementing the often challenging judgment of pulmonary congestion. Adding periodic evaluation of ventricular filling pressures and LUS scores to clinical assessment can optimize treatment decisions and improve patient care. LUS score was a significant predictor for in-hospital and post-discharge clinical outcomes.

**Graphical abstract:**

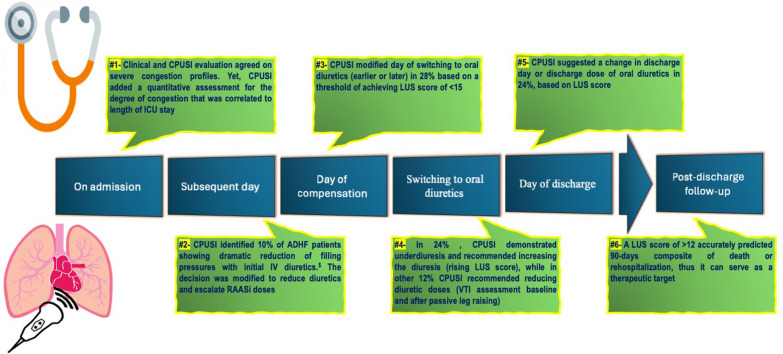

## Background

Heart failure (HF) remains a major health problem, significantly inflating the global health-related costs [1–3]. Despite the critical need for accurate evaluation of fluid status (volume overload vs euvolemia), most of the available tools lack satisfactory sensitivity or specificity or both [4]. The occasional inability to accurately judge congestive status and accordingly optimize treatment decisions was identified as a major contributor to the uncontrolled inflation in HF rehospitalizations (HFH) [5].

Lung ultrasound (LUS) has recently emerged as a new assessment tool for lung congestion through the quantification of B-lines [6–9]. These B-lines represent the reverberation artifacts, originating from water-thickened pulmonary interlobular septa [9]. The diagnostic usefulness of B-lines has been initially identified in intensive care units (ICU) to differentiate pulmonary edema from other causes of acute respiratory distress syndrome (ARDS) [7]. Subsequently, LUS has demonstrated its diagnostic value in identifying a cardiogenic origin of dyspnea in various settings [i.e., outpatients, emergency departments (ED), ICU, and cardiology inpatient units] [8–10]. Additionally, evidence supports that LUS has an important prognostic value in patients with AHF, both on admission and at discharge [11].

In this study, we strived to evaluate the benefits of the systematic addition of cardiopulmonary ultrasound imaging (CPUSI) to conventional management in optimizing ADHF management.

## Methods

This study was a prospective interventional study with a single arm, with a before–after design, evaluating the utility of systematically adding CPUSI to ADHF management. The study was conducted in the Cardiology Department, Cairo University Hospitals, through the period from January 2022 to March 2023. The study protocol was registered and approved by the research ethics committee.

### Study population

The study recruited patients with heart failure with reduced ejection fraction HFrEF hospitalized for acute decompensation with NYHA class III or IV and requiring intravenous (IV) diuretics. Inclusion criteria were: (1) established diagnosis of HFrEF with documented LVEF < 40% for ≥ 3 months; (2) warm–wet phenotype with clinically-overt pulmonary and/or systemic congestion; and (3) age between 18-to-80 years. Exclusion criteria were: (1) refusal to participate; (2) unfeasibility to perform LUS [such as patients with pneumothorax, previous pneumonectomy or lobectomy, or surgically treated lung injuries]; (3) patients with end-stage renal disease (ESRD) or hemodialysis, whose response to diuretic therapies is altered; and (4) patients presenting with cardiogenic shock or requiring upfront invasive mechanical ventilation.

### Study workup

After approval to participate in the study via a written informed consent, recruited patients were evaluated on admission for confirmation of eligibility criteria and tabulation of their baseline clinical data. History taking focused on patient demographics, cardiovascular risk profile, etiology of HFrEF, the potential trigger for the index decompensation, and prior HFH through the past 6 months. Also, the current medications were fully revised (both for HF and other comorbidities). A baseline clinical examination was completely undertaken highlighting blood pressure, heart rate and rhythm, admission weight, lower limbs edema, jugular venous pulse, clinically detected ascites, and/or pleural effusion. Patients’ symptoms and signs of HF were graded according to NYHA and Killip class grading, respectively. Serum creatinine, urea, and electrolytes were checked at baseline and daily or more frequently as appropriate. As per institutional protocol, all HFrEF patients on admission were screened for iron deficiency and thyroid dysfunction.

### Cardiopulmonary ultrasound imaging (CPUSI)

A detailed cardiopulmonary assessment was performed at baseline (within 3 hours (h) from admission), then repeated daily focusing on the changes in ventricular filling pressures and 8-zone LUS score. Both cardiac and pulmonary sonographic examinations were performed using the bed-side echocardiography machine (Philips CX50 Portable Ultrasound system), and the1-4 MHz cardiac probe (Philips S4-1 Ultrasound Transducer), albeit with dedicated lung presets for LUS exam (off tissue harmonics options and optimizing depth and 2D gain to lung tissue). The detailed echocardiographic assessment was performed according to the European Association of Cardiovascular Imaging (EACVI) recommendations [12]. The follow-up echocardiographic assessments focused on the temporal changes of the surrogates for right ventricle (RV) filling pressures (mainly the RV dimensions, the inferior vena cava (IVC) diameter, and its respirophasic changes) and left ventricle (LV) filling pressures (E;’ velocity by tissue Doppler and E/E’ ratio).

Additionally, with continuing diuresis, stroke volume was periodically assessed at a flat supine position, then reassessed after passive leg raising 45° for 3 min (augmenting LV preload by increasing venous return). A significant increase (≥ 15%) in stroke volume after passive leg raising was a marker for volume responsiveness [13] and was considered a red flag for possible overdiuresis. Stroke volume was evaluated by pulsed wave Doppler assessment of the velocity time integral over the left ventricle outflow tract (LVOT) from apical 5-chamber view in the supine position (flat then after leg raising), while LVOT diameter was measured from the left parasternal view in left lateral position during the initial examination.

The focused 8-zone LUS protocol relied on quick screening of the upper and lower of the anterior and lateral lung fields bilaterally, then calculating the B-lines score by summating the space with the maximum number of lines in each of the 8 zones. Generally, according to the LUS score, the degree of pulmonary congestion was categorized into (No-to-mild = 0-to- < 15 lines; Moderate 15-to-30 lines; Severe congestion > 30 lines) [9].

The study protocol entailed completing the clinical judgment and drafting the clinical decision accordingly (specifically decision concerning modifications of diuretics vs. afterload reducing agents: [mainly, renin–angiotensin–aldosterone system inhibitors (RAASi)]. This is followed by performing the CPUSI and revising if it would lead to change/modification of the therapeutic decision. Despite this protocol being performed daily, yet, for avoidance of futile repetition, the investigators opted to perform statistical analysis for the 5 eventful assessments mirroring a significant change in the management course. These 5 assessments were timed as.

- Assessment #1 → Baseline: within 3 h from admission
- Assessment #2 → After 24 h from admission (after initial decongestion)
- Assessment #3 → Day of compensation (de-escalation of IV diuretics)
- Assessment #4 → Day of shifting to oral diuretics
- Assessment #5 → Day of discharge

Daily progress was tabulated including reporting total IV Frusemide in the previous 24h, urinary output, negative balance, body weight, and the follow-up laboratory results. The main clinical outcomes that were sought were the length of ICU stay, length of IV diuretics period, the cumulative dose of IV diuretics, time to compensate to NYHA ≤ 2, and the total length of hospital stay. Also, worsening renal function (WRF) and acute kidney injury (AKI) were meticulously sought and reported. WRF was defined as an increase of ≥ 0.3 mg/dL in the serum creatinine level compared with the value on admission [14]. While AKI (stage I) was defined as either: 1) increase in serum creatinine by ≥ 0.3 mg/dL within 48 h; OR, 2) increase in serum creatinine to ≥ 1.5 times the baseline occurring within the prior 7 days; OR, 3) urine volume < 0.5 mL/kg/h over a 6-h period [15].

After the compensation of HF manifestations, a detailed CPUSI was undertaken and contrasted with the baseline assessment. A threshold of < 15 B-lines score was considered the acceptable target for discharge [16]. Post-discharge, patients were followed up for at least 90 days to report death or HFH.

### Statistical analysis

Statistical package for social science (SPSS) software for Microsoft Windows, version 22 (SPSS Inc., Chicago, IL, USA) as used for data analysis. Categorical data were presented as frequency and percentages (*n* (%)), and correlations among them were analyzed by Chi-square test. Continuous data were checked for normality using the Shapiro–Wilk test and were presented as mean (± standard deviation (SD)) or median (interquartile range (IQR)) as appropriate. Continuous data were analyzed using one-way analysis of variance (ANOVA). Because of the “before-and-after design,” Mc-Nemar and repeated ANOVA tests were utilized to evaluate the differences in patients’ assessments through the different study checkpoints. A receiver operating characteristic (ROC) curve analysis was performed to identify the predischarge LUS score that effectively predicts post-discharge death or HFH. A probability *p*-value less than 0.05 was considered statistically significant.

## Results

**Fig. 1:**
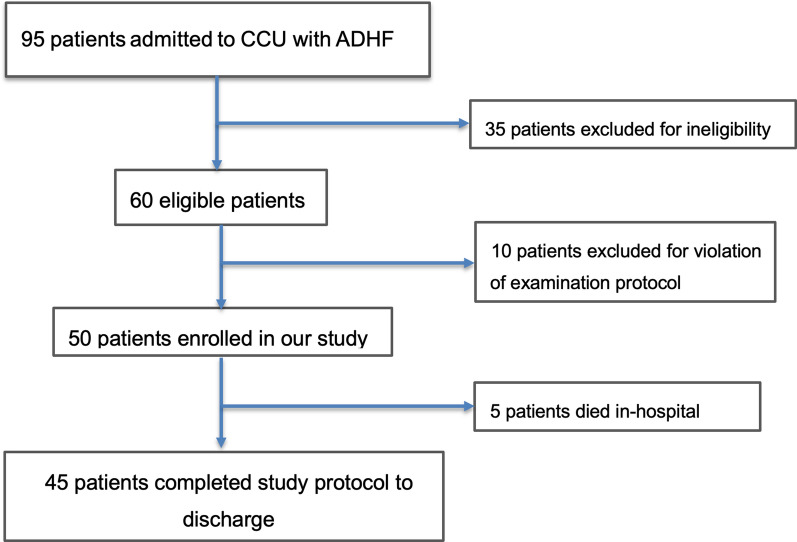
Flow chart demonstrating the study recruitment process

Among 95 ADHF patients screened, 35 patients were excluded for ineligibility. The 60 eligible patients were recruited to the study, yet 10 of them had their management (according to the consultant physician) violating the study protocol, and thereby were excluded from the analysis. The remaining 50 patients comprised the study group who completed the protocol throughout the hospital course. There were 5 in-hospital mortality cases. The patients’ recruitment process is highlighted in Fig. 1. The mean age of the study group was 5.7 ± 10.59 years, with male gender representing 80% of the study cohort. Other baseline clinical and laboratory characteristics of the study group are demonstrated in Table 1, while data of the baseline CPUSI is demonstrated in Table 2.

**Table 1:** Baseline clinical and laboratory assessments for the study group

Age (years)	55.72 ± 10.59
Male gender *n* (%)	40 (80%)
Active smoker	16(32%)
Diabetes mellitus	31(62%)
Hypertension	33(66%)
**Etiology of HF**	
Ischemic	36(72%)
Valvular	7(14%)
Idiopathic	3(6%)
Post-myocarditis	3(6%)
Hypertensive	1(2%)
Triggers to decompensation	17(34%)
Non-compliance to therapy	16(32%)
Infection	5(10%)
Ischemic insult	9(18%)
Tachyarrhythmia	2(4%)
Brady arrhythmia	1(2%)
**Prerecruitment pharmacologic therapy**	
Patients on beta-blockers	33(60%)
Beta-blockers dose equivalent	50% (25–50)
Patients on RAASi	44(80%)
RAASi dose equivalent	62.5% (25–100)
Patients on MRA	41 (82%)
MRA dose equivalent	63.64 ± 22.79
Patients on SGLT2i	13 (26.0%)
Patients on digoxin	5 (10.0%)
**Physical examination**	
Systolic BP < 90	4 (8.0%)
Atrial fibrillation	12 (24.0%)
Admission weight (Kg)	88.15 ± 18.30
JVP (cm above clavicle in 45°)	19.78 ± 3.23
Ascites	14 (28.0%)
Pleural effusion	18 (36.0%)
**NYHA classification**	
NYHA 3	24 (48.0%)
NYHA 4	26 (52.0%)
**Killip class**	
Killip class 2	16 (33.0%)
Killip class 3	29 (58.0%)
Killip class 4	5 (9.0%)
**Laboratory workup**	
Serum creatinine (mg/dl)	1.21 ± 0.23
Serum urea (mg/dl)	59.7 ± 28
Serum sodium (Na) (mEq/L)	132.6 ± 10.2
Serum potassium (K) (mEq/L)	4.1 ± 0.6
Hemoglobin (gm/dl)	11.96 ± 1.91
Serum albumin (gm/dl)	3.49 ± 0.45
Transferrin saturation (%)	15.2 ± 9.1
Serum ferritin (µg/dL)	25 (15–120)
TSH (IU/ml)	3.1 (2.1–5.4)

Data represented as frequency (percentage), mean ± standard deviation or median (interquartile range) as appropriate

BP, Blood pressure; HF, Heart failure; JVP: Jugular venous pulse; MRA, Mineralocorticoid antagonist; NSAID, Non-steroidal anti-inflammatory drugs; NYHA, New York Heart Association; RAASi, Renin–angiotensin–aldosterone system inhibitors; SGLT2, Sodium–glucose co-transporter 2 inhibitors; TSH, Thyroid stimulating hormone

**Table 2:** Cardiopulmonary sonographic imaging data within 3 h of admission

Left ventricular end-diastolic diameter (LVEDD) cm	6.09 ± 0.62
Left ventricular end-systolic diameter (LVESD) cm	5.26 ± 0.56
Left ventricular ejection fraction (LVEF) %	26.18 ± 7.40
Left atrium diameter (LA) cm	4.71 ± 0.49
Mitral regurgitation (MR) moderate or severe	29 (58%)
E/A ratio	2.79 ± 0.52
E velocity (cm)	96.17 ± 15.19
Medial E' velocity (cm/s)	5.51 ± 2.13
Lateral E' velocity (cm/s)	7.58 ± 3.28
E/E' medial	19.82 ± 8.33
E/E' lateral	14.59 ± 6.39
E/E' average	18.36 ± 6.85
Left ventricular outflow tract (LVOT) area (cm^2^)	3.39 ± 0.98
Right ventricle basal dimension (cm)	4.59 ± 0.55
Right ventricle mid-cavity dimension (cm)	4.60 ± 0.95
Right ventricle longitudinal dimension (cm)	7.91 ± 0.75
TAPSE (cm)	1.35 ± 0.29
IVC inspiratory diameter (cm)	2.06 ± 0.33
IVC expiratory diameter (cm)	2.43 ± 0.23
8-zone LUS score	56 (48–62)

Data represented as frequency (percentage), mean ± standard deviation or median (interquartile range) as appropriate

IVC, Inferior vena cava; LUS, Lung ultrasound; TAPSE, Tricuspid annular plane systolic excursion

As per study protocol, the standard of care clinical judgment was completed first, then the CPUSI was pursued to assess if it recommended change/modification of the therapeutic decisions. Although this protocol was performed daily, yet as previously explained comparative analyses were highlighted on the 5 landmark points in ADHF management, timed by: baseline, after 1 day from admission, day of compensation, day of switching to oral diuretics, and lastly day of discharge. As such, we analyzed 236 pairs of clinical evaluation and CPUSI to highlight the clinical implications of adding LUS and echocardiographic assessments to the standard clinical evaluation.

- On admission, (Assessment #1), nearly all patients were characterized by markedly elevated RV- and LV-filling pressures and severely congested lung fields by LUS. IV diuretics were the mainstay of initial therapy as agreed between the clinical- and CPUSI judgment. However, the CPUSI helped to provide a quantitative evaluation of the magnitude of pulmonary congestion (baseline filling pressures and LUS score).
- On the following day of admission (Assessment #2), CPUSI modified decisions in 5 patients (10%), voting for reducing the diuretic doses and uptitration of the afterload reducing agents (RAASi). These patients had significant improvement in the number of B-lines compared to baseline assessment. Their median baseline LUS score was 50 and became 24 the next day with a *p* < 0.0011. Conceivably, these patients represent the subgroup of ADHF more likely to be decompensated due to volume redistribution rather than volume overload.
- On the day of compensation (Assessment #3), in 14 patients (28%) CPUSI modified the day to reduce the IV diuretic dose (either earlier or later) different than clinical judgment. LUS decision was essentially based on achieving a threshold of LUS score < 15 B-lines.
- On the day of switching to oral diuretics (Assessment #4), in 12 patients (24%) the CPUSI suggested continuing diuresis for inadequate pulmonary decongestion, while in 6 other patients (12%), it suggested lowering the diuretic dose because the PWD evaluation of the stroke volume at rest and after 3 min of passive leg raising suggested overdiuresis.
- On the day of discharge (Assessment #5), the CPUSI judged 11 patients (24%) to be inadequately decongested suggesting a change in the diuretic dose or the time of discharge.

**Fig. 2:**
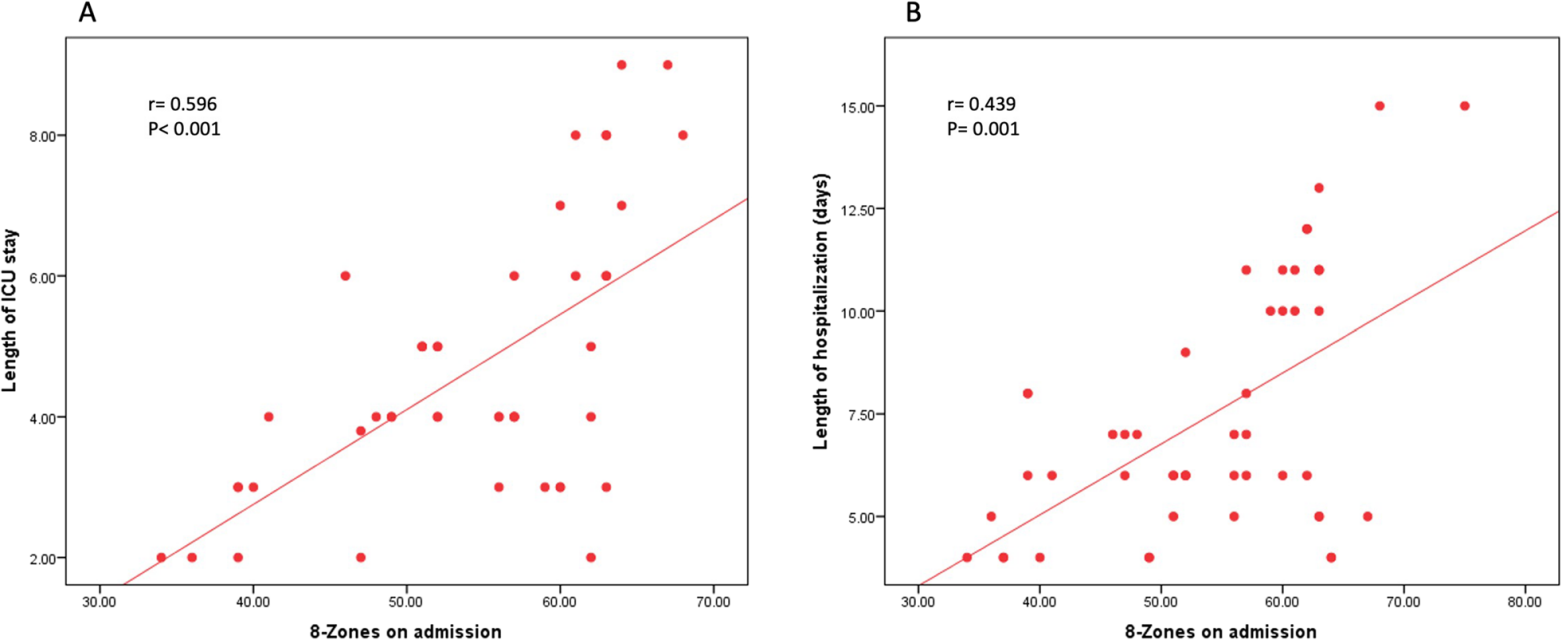
Correlation of 8-zone LUS score on admission with length of ICU stay (**A**) and total length of hospitalization (**B**)

Collectively, adding CPUSI to the standard of care clinical judgment has modified clinical decisions and improved patient care in 57 out of 236 assessments (24%). The temporal changes of the CPUSI parameters through these 5 landmark assessments are demonstrated in Table 3. Also, the LUS score on admission was found to be correlated to the length of ICU stay and the whole length of hospitalization, with a correlation coefficient *r* = 0.596 and = 0.439, respectively (*p* < 0.001 for both) (Fig. 2).

**Table 3:** Comparative CPUSI assessments across the study checkpoint

*Combined sonographic assessment through landmark examinations						
	Assessment 1	Assessment 2	Assessment 3	Assessment 4	Assessment 5	Test value
E/E' average	18.36 ± 6.85	16.59 ± 5.96	13.39 ± 5.73	12.19 ± 5.25	12.81 ± 4.63	23.083•
IVC (inspiration)	2.06 ± 0.33	1.63 ± 0.53	1.37 ± 0.50	1.44 ± 0.42	1.12 ± 0.41	25.185•
IVC (expiration)	2.43 ± 0.23	2.19 ± 0.32	1.88 ± 0.34	1.82 ± 0.40	1.76 ± 0.16	48.648•
8-zone LUS score	56 (48–62)	44 (25–55)	14 (9–24)	11 (6–14)	6 (3–10)	60.350‡

*Comparative statistics for CPUSI serial evaluations to baseline evaluation						
		Assessment 2 Vs Assessment 1	Assessment 3 Vs Assessment 1	Assessment 4 Vs Assessment 1	Assessment 5 Vs Assessment 1	
E/E' average >		1.000	< 0.01	< 0.01	0.002	
IVC (inspiration)		0.001	0.002	< 0.01	< 0.01	
IVC (expiration)		< 0.01	< 0.01	< 0.01	< 0.01	
8-zone LUS score		< 0.01	< 0.01	< 0.01	< 0.01	

^*^Repeated Measure ANOVA test; ‡: Friedman test

Assessment #1 = Baseline: within 3 h from admission; Assessment #2 = After 24 h from admission (after initial decongestion); Assessment #3 = Day of compensation (de-escalation of IV diuretics); Assessment #4 = Day of shifting to oral diuretics; Assessment #5 = Day of discharge; LUS, Lung ultrasound; IVC, Inferior vena cava

Throughout the hospital course, kidney functions were regularly checked to monitor the anticipated rise in serum creatinine with IV diuresis. Threshold to diagnose WRF was achieved in 25 (50%), while AKI occurred in 9 (18%) of the study group. None of the patients required dialysis or ultrafiltration. The mean length of ICU stay was 4.6 ± 1.9 days, while the mean time of IV diuretics (till switching to oral) was 4.9 ± 2.1 days with a median cumulative IV Frusemide dose of 440mg (340–720).

By the time of discharge, cumulative weight loss since admission was 10.2kg ± 4.7, 31 patients (62%) were judged as NYHA class 2, while 14 (28%) as class 1. The median oral diuretic discharge dose was 10mg (10–20) of torsemide. The contrast between the comprehensive on-admission and predischarge CPUSI evaluations is detailed in Table 4.

**Table 4:** Contrasting the CPUSI parameters between admission and predischarge

	On admission (*n* = 50)	On discharge (*n* = 45)	*p* value
Left ventricular end-diastolic diameter	6.09 ± 0.62	5.94 ± 0.57	**0.019**
Left ventricular end-systolic diameter	5.26 ± 0.56	5.45 ± 2.10	0.463
Left ventricular ejection fraction (LVEF) %	26.18 ± 7.40	27.55 ± 7.88	0.057
Left atrium diameter (LA) cm	4.71 ± 0.49	4.59 ± 0.59	**0.039**
Mitral regurgitation (MR) moderate or severe	29 (58%)	26 (54%)	0.982
E/A	2.79 ± 0.52	2.41 ± 1.74	0.165
E velocity	96.17 ± 15.19	75.99 ± 20.61	** < 0.001**
Medial E'	5.51 ± 2.13	6.99 ± 3.09	** < 0.001**
Lateral E'	7.58 ± 3.28	9.10 ± 3.65	**0.001**
E/E' medial	19.82 ± 8.33	12.86 ± 5.26	** < 0.001**
E/E' lateral	14.59 ± 6.39	10.72 ± 4.18	**0.001**
TR. Vmax	2.93 ± 0.59	3.01 ± 0.60	0.064
Right ventricle basal dimension (cm)	4.59 ± 0.55	4.25 ± 0.69	** < 0.001**
Right ventricle mid-cavity dimension (cm)	4.60 ± 0.95	4.57 ± 1.13	0.423
Right ventricle longitudinal dimension (cm)	7.91 ± 0.75	7.21 ± 2.09	**0.007**
TAPSE	1.35 ± 0.29	1.30 ± 0.44	0.451
IVC inspiratory diameter (cm)	2.06 ± 0.33	1.12 ± 0.41	** < 0.01**
IVC expiratory diameter (cm)	2.43 ± 0.23	1.76 ± 0.16	** < 0.01**
8-zone LUS score	56 (48–62)	6 (3–10)	** < 0.01**

Bold indicates statistically significant *p* values

Data represented as frequency percent or mean ± standard deviation as appropriate

IVC, Inferior vena cava; TAPSE, Tricuspid annular plane systolic excursion; TR. Vmax, Tricuspid regurgitation maximum velocity

Through the follow-up period of 90 days, the composite of death or HFH occurred in 4 patients (9%), with a total of 4 HFH events including 1 mortality.

**Fig. 3:**
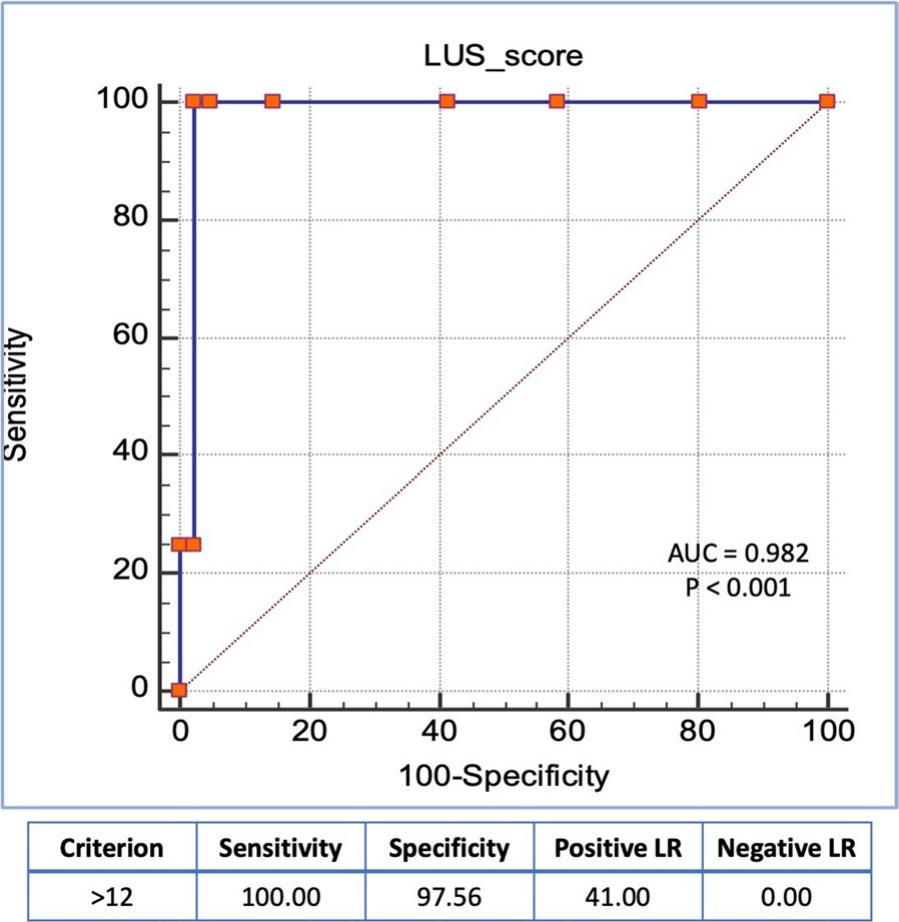
Receiver operating characteristic (ROC) curve analysis for the prediction of predischarge LUS score to HF rehospitalization and death through 3 months

The predischarge LUS score was found to be a significant predictor for the composite of death or HFH. In a receiver operating characteristics (ROC) curve analysis, a LUS score > 12 had a sensitivity of 100% and a specificity of 98% for the composite of death or HFH through the 90 days post-discharge (Fig. 3).

**Fig. 4:**
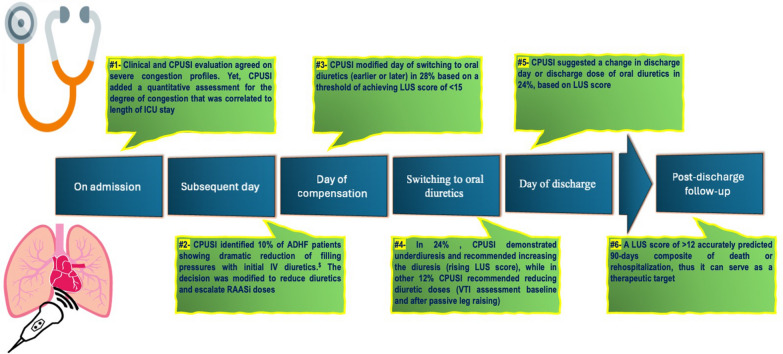
Additive role of CPUSI in ADHF management through the study checkpoints. ^$^likely decompensated for volume redistribution rather than volume overload. ADHF: Acute decompensated heart failure; CPUSI: Cardiopulmonary ultrasound imaging; LUS: Lung ultrasound; RAASi: Renin–angiotensin–aldosterone system inhibitors; VTI: Velocity time integral

The additive beneficial role of CPUSI throughout the study checkpoints as adjunctive to clinical assessment is summarized in Fig. 4.

## Discussion

This study prospectively enrolled 50 ADHF patients (warm and wet), to evaluate the additive benefit of systematic CPUSI to optimize management decisions. Added to clinical assessment, CPUSI modified treatment decisions in 57 out of 236 examinations (24%) through various stages of ADHF management. Also, 8-zone LUS proved to be a significant prognosticator, where the B-lines score on admission was strongly correlated to the length of hospital stay, while the predischarge score precited the composite of death or HFH through the subsequent 90 days.

HF remains one of the major problems burdening national health systems [1–3]. Thanks to the improving longevity worldwide and the improved survival of many cardiovascular diseases, the prevalence of HF is growing to nearly 64 million people, with a lifetime risk of 20% of adults beyond the age of 40 years [17]. The bigger cut of the HF-related expenditure goes for the high rate of recurrent hospitalizations, almost reaching 50% of the discharged patients within the subsequent 6 months [1]. The gradual accumulation of intravascular and interstitial fluid is the main cause of the clinical presentation by ADHF and requiring rehospitalization [18]. Thereby, signs and symptoms of pulmonary and/or systemic congestion are the most prevailing presentation encountered in the majority of ADHF patients presenting to the ED [4, 5].

Moreover, it was identified that discharging HF patients with residual congestion (incompletely diuresed) is linked to an increased risk of subsequent rehospitalization, probably because it precipitates faster recurrent congestion [16]. Hence, it seems that accurate assessment of the volume status in HF patients is a critical step in optimizing patients’ care. Early identification of gradual fluid accumulation in ambulant patients can certainly prevent a lot of outpatients from worsening to the point of ADHF requiring hospitalization. Similarly, optimizing fluid status by the time of discharge would help in reducing rates of HFH. Surprisingly, overdiuresis was proved as well to be linked to increased rehospitalization, presumably, due to the subsequent neurohormonal and hemodynamic derangements that it can trigger [4, 19]. This fact adds to the importance of meticulous and accurate judgment of the volume status in HF patients to guard against under- or overdiuresis.

Despite critically needed, pooled evidence indicates that accurate assessment of the degree of congestion (amount of overload) is often challenging and is often more puzzling in pulmonary congestion compared to systemic congestion [4]. Conventionally, the clinical assessment of left-sided (pulmonary) congestion had been primarily based on major and minor HF Framingham’s criteria, orthopnea, dyspnea, pulmonary rales, and X-ray evidence of lung congestion or pleural effusion, however, all are plagued by modest sensitivity or specificity or both [4]. Hence, in daily practice, occasionally accurate assessment of volume status to guide decongestive therapy is really challenging.

LUS has emerged as a semiquantitative method to assess pulmonary congestion in HF patients [10]. A combined protocol including LUS and echocardiography was originally suggested under the name of cardiothoracic ultrasound (CaTUS) by Öhman et al., in 2019, and provided excellent accuracy for diagnosing AHF in ED [20]. They examined 100 patients presenting with undifferentiated acute dyspnea to the ED by the CaTUS protocol upon arrival. CaTUS was considered positive for AHF when E/E’ was > 15 and there were significant bilateral B-lines or bilateral pleural fluid, on LUS. All 100 patients were sampled for brain natriuretic peptide (BNP), and 96 patients underwent chest radiography in the ED, which was analyzed afterward by a blinded radiologist. The reference diagnosis of AHF consisted of elevated BNP in combination with congestion on chest radiography. CaTUS had a sensitivity of 100% and a specificity of 95.8% in diagnosing AHF in ED with diagnostic accuracy higher than either E/E’ or LUS alone [20].

Unfortunately, there is a lack of standardization of LUS interpretation, ranging between qualitative (A vs. B profile), semiquantitative (positive if ≥ 1 bilateral zone with > 3 lines), and quantitative (total sum of B-lines in the examination) [9]. However, Picano’s congestion grading remained among the most supported classifications: mild (6–15 B-lines), moderate (16–30 B-lines), and severe: > 30 B-lines [9].

Despite the huge additive value for LUS in cardiac units, its utilization remained limited in real-world daily practice. The main barriers to systematizing utilization of LUS in cardiac wards and intensive care units seemed to be: 1) appreciating a long time for a comprehensive 28-zone LUS protocol, and 2) lack of awareness about the ease of performing the exam. Therefore, it is presumable that if cardiologists are trained to perform a time-efficient 8-zone LUS examination by the same cardiac probe, it can be of extreme value in improving decision-making and therapeutic strategies in ADHF patients.

In this study, we performed 236 CPUSI on 50 patients hospitalized for ADHF with warm–wet phenotype. The mean age was 55.7 ± 10.6 years, and 80% of the study group were males. In our study cohort, 28 patients (56%) reported prior ADHF hospitalization within the past 6 months.

On admission, both comprehensive clinical judgment and the CPUSI agreed that patients were significantly congested (volume overloaded), yet CPUSI had the merit of providing quantitative measures that served as a baseline for subsequent assessments. Additionally, LUS score on admission was strongly correlated to the length of hospitalization, where patients with higher LUS scores had longer stays in the ICU and total length of hospital duration.

Subsequent evaluations of the surrogates of LV-, RV-filling pressures, and the LUS score were useful and measurable tools to monitor the dynamicity and guide the progression of decongestion therapy. Through various stages of management, adding CPUSI to the clinical judgment modified therapeutic decisions and helped to improve patients’ care.

On day 2 of hospitalization, CPUSI identified patients with mainly volume redistribution (rather than volume overload), voting for the reduction of diuretics and uptitration of afterload reducing agents (mainly RAASi). In the subsequent days, CPUSI proved quite helpful to monitor the rate of decongestion and to judge the ultimate time for IV diuretics to reduce the dose or to switch to oral. Assessment of stroke volume by PWD over the LVOT at rest then repeated after 3 min of leg raising was systematically performed to identify patients likely to have been over-diuresed, so their diuretic doses were reconsidered. Lastly, and most importantly, LUS was utilized to ensure appropriate normalization of the volume status at predischarge status. A stable B-line score ≤ 15 was considered the threshold to define adequate pulmonary decongestion.

Additionally, this study allowed the physicians to monitor the temporal improvements in the RV- and LV-filling pressures and LUS lung scores paralleling the improvement in NYHA class during the period of hospitalization. It was quite remarkable to observe E/E’ ratio declining steadily from 18.4 ± 6.8 to 12.8 ± 4.6, the IVC diameter marching from 2.4 ± 0.2 to 1.76 ± 0.2 cm, and the LUS score falling from 56 (48–62) to 6 (3–10) through the serial assessments from admission to predischarge.

Similar observations for the dynamics of echocardiographic and LUS parameters through decongestive therapy were reported in other studies [11, 21]. In a cohort of 340 patients admitted for dyspnea, Frassi et al. showed that in the subgroup (*N* = 70) exhibiting a clinical response to treatment (i.e., a decline in NYHA class by ≥ 1), the B-line count (assessed with the 28-zone protocol) was significantly and steadily decreasing from baseline to discharge (42 ± 32 vs. 15 ± 18, *p* <  < 0.0011) [21]. Likewise, changes in B-line counts were reported in a small cohort of 25 ED patients after 24 h of IV diuretic therapy (53 ± 17 vs. 32 ± 14, *p* < 0.001) [22]. Overall, it seems very plausible that B-line clearance during pulmonary decongestion therapy is significantly and very closely correlated with improvement in dyspnea, NYHA class, and various examination findings [10, 11].

In this study, we have identified a threshold of > 12 B-lines by ROC curve analysis to predict rehospitalization, with excellent diagnostic accuracy. A similar cutoff (> 12 B-lines) was found to be associated with rehospitalization in other studies [23]. Other studies had identified that > 15 B-lines on predischarge evaluation were associated with higher BNP levels, higher LV-filling pressure estimates, and higher rates of HFH [16, 24, 25]. However, in the majority of these studies the time of discharge was clinically decided, in contrast to our design which considered < 15 B-lines as a threshold for complete compensation and safe discharge. Also, many of these studies utilized the 28-zone LUS protocols, which can give higher scores than the 8-zone protocol and many of them were recruiting HF with both reduced- and preserved-ejection fraction (HFrEF and HFpEF, respectively).

Thereby, we presume that a threshold of ≤ 12 B-lines can be a safer and better marker for adequate decongestion in the management of decompensated HFrEF patients, striving to reduce the rates of early HFH. The relatively lower rate of HFH in our cohort (9%), compared to the 14–18% in the formerly mentioned studies [16, 25], is in favor of the lower LUS score. More evidence would be recommended to confirm if a predischarge LUS score of ≤ 12 lines should be considered the ultimate target in ADHF management.

In summary, we have found that systematic CPUSI provided an excellent addition to the standard care ADHF management. Including the focused 28-zone LUS protocol made the CPUSI very handy and time-efficient and was not overburdening the treating physicians. Diagnostic accuracy of the 8-zone LUS score was notable through the different management stages from admission to discharge. LUS score showed excellent predictive ability for the length of hospital stay and subsequent HFH by the on-admission and the predischarge assessments, respectively.

### Clinical implications

This exploratory study paves the way to include CPUSI as an adjunctive tool to daily ADHF management. CPUSI proved to be a practical, easy to learn, fast to perform, and quite time- and resources-efficient tool in optimizing therapeutic strategies and improving outcomes for HF patients.

### Limitations

This study had certain limitations to mention. First, it is a single-arm study based on a “before-and-after” design, aiming to appreciate the change in management strategies after CPUSI. We considered this as an exploratory study, to praise CPUSI as an easy, fast, and practical tool that can be implemented in daily ADHF management to improve patients’ outcomes without excess burden on the resources. Also, CPUSI operators were not blinded to patients’ clinical data, which theoretically could allow for interpretation bias. The limited time of follow-up to 90 days might have led to underestimation of the rate of hospitalization, hence, longer follow-ups are recommended in future studies to emphasize CPUSI's full impact on outcomes. The unavailability of routine BNP assessment on admission and predischarge is another limitation that was imposed because of logistics and funding restrictions. Finally, because only 4 patients experienced the composite endpoint through the post-discharge period, the reported cutoff should be interpreted with caution. Another larger, 2-arms, randomized study to endorse the advantage of systematic CPUSI in ADHF management would be highly recommended to confirm these findings.

## Conclusion

In ADHF patients, adding systematic CPUSI to clinical assessment can improve management strategies and patients’ outcomes. CPUSI, by providing timely monitoring of biventricular filling pressures and LUS score, an significantly improve the accuracy of evaluating congestive status. LUS score also was a significant predictor for in-hospital and post-discharge outcomes.

## Data Availability

These can be made available upon reasonable request from the corresponding author.

## Abbreviations

ADHF: Acute decompensated heart failure
AHF: Acute heart failure
AKI: Acute kidney injury
ARDS: Acute respiratory distress syndrome
BNP: Brain natriuretic peptide
CaTUS: Cardiothoracic ultrasound
CPUSI: Cardiopulmonary ultrasound imaging
EACVI: European Association of Cardiovascular Imaging
ED: Emergency departments
ESRD: End-stage renal disease
HF: Heart failure
HFH: Heart failure rehospitalization
HFpEF: Heart failure with preserved-ejection fraction
HFrEF: Heart failure with reduced ejection fraction
ICU: Intensive care units
IV: Intravenous
IVC: Inferior vena cava
LUS: Lung ultrasound
LV: Left ventricle
LVEF: Left ventricular ejection fraction
LVOT: Left ventricular outflow tract
NYHA: New York Heart Association
PWD: Pulsed wave Doppler
RAASi: Renin–angiotensin–aldosterone system inhibitors
ROC: Receiver operating characteristics
RV: Right ventricle
WRF: Worsening renal function
